# When a mean can be meaningless: evaluating mosquito infections with *Plasmodium* parasites

**DOI:** 10.1017/S0031182025100541

**Published:** 2025-08

**Authors:** Prince Chigozirim Ubiaru, Lisa C. Ranford-Cartwright

**Affiliations:** School of Biodiversity, One Health and Veterinary Medicine, College of Medical, Veterinary and Life Sciences, University of Glasgow, Glasgow, Scotland, UK

**Keywords:** glm, infection prevalence, logistf, malaria, mosquito infection, oocyst intensity

## Abstract

Several malaria control measures aim to reduce infection levels in mosquitoes, and evaluation of these measures usually relies on experimental infections of mosquitoes or evaluation in field populations. Both require robust statistical tools to account for multiple variables and non-normal distributions of parasites in the vector host. We argue that a well-chosen generalized linear or mixed model is the most appropriate statistical tool for analysing and interpreting these biological data. We suggest specific methods to overcome datasets where some groups have zero/close to zero prevalence, or many zero counts of parasite numbers (as would be seen with an effective transmission blocking intervention). These methods are more broadly applicable across many parasitic infections with similar patterns of parasite numbers across hosts.

## Graphical abstract

**Figure:**
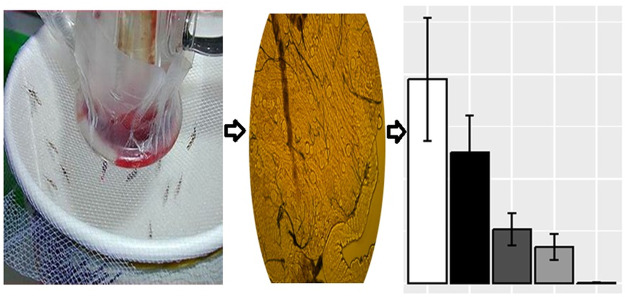

## Introduction

Malaria is one of the most severe and life-threatening tropical diseases, affecting nearly half of the world’s population; in 2023 there were 263 million cases globally, and 597,000 deaths (World Health Organisation, 2024). People are infected through the bite of an infected female *Anopheles* mosquito. The disease can be treated by chemotherapy, usually with an Artemisinin-based combination therapy for the most pathogenic species *Plasmodium falciparum*, and for relapsing species like *Plasmodium vivax*, also with primaquine (World Health Organisation, 2024). Public health control strategies include intermittent preventive therapy and vector control, employed to reduce the disease burden significantly in malaria-endemic areas. However, these control strategies are challenged by parasite drug resistance (reviewed in Ippolito et al., 2021) and mosquito insecticide resistance (reviewed in Suh et al., 2023).

Currently, many malaria control measures that are being developed aim to reduce the proportion of mosquitoes in a population that are capable of transmitting malaria. Examples include (i) interventions that aim to increase the refractoriness of mosquitoes to malaria parasites by genetic modification, paratransgenesis or alterations in mosquito microbiota (reviewed in Kefi et al., 2024), like the use of a gene drive system within the mosquito vector to reduce and/or prevent the parasite’s survival and transmission; (ii) transmission-blocking drugs and vaccines (reviewed in Yu et al., 2022); and (iii) interventions that aim to reduce the age distributions of vector mosquitoes below the extrinsic incubation period (EIP). The EIP is the time it takes for *Plasmodium* parasites to develop, replicate and invade a mosquito’s salivary glands after she takes an infected blood meal. For example, the EIP of *P. falciparum* is between 12 and 14 days (Vaughan et al., 1992; Ohm et al., 2018), so for *P. falciparum* transmission to occur in humans, mosquito vectors must live beyond the EIP (Shaw et al., 2020).

To evaluate the success of these control measures, it is necessary to compare the level of mosquito infection in a population before and after the intervention, or in experimental work, between control and treated mosquitoes given infectious blood meals. These studies require proper statistical models or tools that incorporate all the different measured factors to estimate the results, which are mainly presented as infection prevalence and oocyst intensity. *Plasmodium*-mosquito infection data, in common with other biological data with multiple measurements or experimental variables, are inherently ‘noisy’ natural systems (reviewed in Paterson and Lello, 2003). These types of data are usually complex, and require well-chosen statistical tools to fit and interpret them. Analysing these data types requires knowledge of the correct statistical tool to use, which is not usually simple. This often results in choosing a more straightforward statistical method to analyse the data, which may not be wholly appropriate.

## Approaches for measuring mosquito infection

Experimentally, the impact of a control measure such as a transmission-blocking drug or antibody on mosquito infection uses 1 of 3 different methods: a direct membrane feeding assay (DMFA), a direct skin feed (DSF), or a standard membrane feeding assay (SMFA). In DMFA, blood containing transmission stages (mature gametocytes) from a treated human host is fed to female *Anopheles* mosquitoes using a water-jacketed membrane feeder (Ponnudurai et al., 1987), whereas in DSF, female *Anopheles* mosquitoes are directly placed on the skin of a gametocyte carrier to feed (Bousema et al., 2012). In SMFA, the gametocytes are grown *in vitro* and fed to *Anopheles* mosquitoes using water-jacketed membrane feeders. DMFA and DSF are usually used in the field to check for the infectiousness of natural gametocyte carriers treated with, for example, potential transmission-blocking drugs. In contrast, SMFAs are used mainly for two purposes: first, to check for the effect of immune factors or transmission-blocking compounds in preventing the transmission of *in vitro* cultured mature gametocytes; second, to study the ability of different clones or isolates of *P. falciparum* to transmit to different *Anopheles* mosquitoes.

The resulting infection for SMFA (for the effect of immune factors/compounds), DMFA and DSF is characterized as transmission-reducing activity (TRA) and transmission-blocking activity (TBA) (Medley et al., 1993; Churcher et al., 2012). TRA is the percentage inhibition in parasite oocyst intensity of infected mosquitoes, whereas TBA is the percentage inhibition in oocyst prevalence (Churcher et al., 2012; Miura et al., 2016). However, there has been debate about using TRA or TBA as the best readout to report for SMFA (Wu et al., 2015; Miura et al., 2016; Swihart et al., 2018).

The result of mosquito infections from membrane feeding assays (MFAs) for any SMFA studies are commonly presented as infection prevalence and oocyst infection intensity; the former is the percentage (%) of mosquitoes infected, usually presented as a mean percentage of replicates, and the latter is the number of oocysts present on mosquito midguts (commonly known as parasite abundance), usually presented as a mean, a geometric mean, or median oocyst number, and occasionally presented for infected mosquitoes only (more properly parasite intensity). The standard membrane-feeding assay additionally includes variables such as the different batches of human serum used for the parasite culture and blood used for setting up the gametocyte cultures. All 3 assays include variables such as the size of each mosquito, the gametocyte number in the blood, and replicate effects, which could all influence the distribution of infection (Ponnudurai et al., 1989; Lyimo and Koella, 1992).

## Common methods for analysing *P. falciparum* mosquito infection data

A survey of papers from PubMed, filtered from the years 2009–2025 and presenting data on mosquito *P. falciparum* infection, showed that, out of 153 published papers reviewed, 78 (∼51%) either used one or a combination of the following tests to analyse their oocyst infection prevalence and intensity data: Chi-squared test, Student’s *t*-test/analysis of variance (ANOVA), Mann–Whitney test, Wilcoxon-signed rank, Kruskal–Wallis/Dunn test and Kolmogorov–Smirnov test (Table 1).

**Table 1.** Summary of statistical tests used for analysing *P. falciparum* mosquito infection between 2009 and 2025 in 153 published papers

Statistical test	Infection prevalence/TBA	Oocyst intensity/TRA
Chi-squared/Fisher’s exact	18	4
ANOVA/Student’s *t*-test	4	9
Mann–Whitney	13	28
Wilcoxon rank-sum/Wilcoxon signed rank	10	12
Kruskal–Wallis/Dunn Test	9	23
Kolmogorov–Smirnov test	–	1
Logistic regression/GLM	13	4
GLMM (zero-inflated negative binomial, zero-truncated Poisson, hurdle model, hurdle negative binomial)	19	61

Publications obtained from PubMed using the search terms ‘membrane feeding’ and ‘falciparum.’ TBA, transmission blocking activity; TRA, transmission-reducing activity.

Although improved mathematical methods/models for analysing mosquito infection data from an MFA have been published (Churcher et al., 2012; Miura et al., 2013; Swihart et al., 2018), many researchers analysed mosquito oocyst infection prevalence and intensity with statistical tests that used mean and median estimates only to analyse mosquito infection data. The use of tests based solely on mean or median values are often inadequate to reveal differences in mosquito infection levels, which could lead to poor interpretation of the overall result. The advantages and limitations of these statistical tests are summarized in Table 2.

**Table 2.** Advantages and limitations of common metrics used for analysing *P. falciparum* mosquito infection prevalence and oocyst intensity

Statistical test	Type of data	Main features/ Advantages	Limitations/ Disadvantages
Chi-squared	Infection prevalence	i. Used to check for a statistically significant difference/ association between two or more categorical variables in a dataset. ii. Does not depend on the assumption of normal distribution of data. iii. Easy to compute and interpret the result.	i. Needs a large data sample size. ii. Unsuitable for continuous data except when grouped into categories. iii. The method used to group the dataset into categories can influence the test result.
Analysis of variance (ANOVA)	Oocyst infection intensity	i. Used to check for differences of means using variance to determine any statistically significant difference between multiple categorical groups simultaneously. ii. Suitable for observing interactions among one or more independent variables in a dataset. iii. Suitable for analysing datasets with repeated observations. iv. Available for use in different statistical software.	i. Each group in the dataset must be normally distributed. ii. The variance among the groups in the dataset should be equal. iii. Not suitable with a small sample dataset. iv. Not easy to interpret the result if there are many variables or interactions.
Mann–Whitney *U*/Wilcoxon rank-sum/signed rank	Infection prevalence and oocyst intensity	i. Uses mainly the median to analyse differences between two independent groups. ii. Suitable for skewed data or data that are non-normally distributed iii. Easy to understand without needing advanced knowledge of statistics and tools. iv. Accommodates a small sample size.	i. Not suitable for comparing more than two independent groups. ii. Assumes two independent groups share the same distribution shape. iii. Cannot give a direct measure of effect size
Kruskal–Wallis	Infection prevalence and oocyst intensity	i. Uses mainly the median to analyse differences among three or more independent groups. ii. Suitable for skewed data and does not require the data to be normally distributed. iii. Flexible to use as it does not assume the same variance among independent groups. iv. Can be used to analyse any data that can be ranked. v. Available for use in different statistical software.	i. Cannot identify the individual variation within each independent group. ii. Not suitable with samples with replicates or matched pairs. iii. Not suitable for handling different variables interaction.
Dunn test	Infection prevalence and oocyst intensity	i. Used to check for statistically significant differences among pairs of group means or medians. ii. Used to analyse any data that can be ranked. iii. Used after carrying out the Kruskal–Wallis test. iv. Suitable for data with non-normal or ordinal distribution with small sample sizes.	i. Computationally expensive when calculating data with many pair groups. ii. The data across the pair groups must be independent. iii. Cannot determine any interaction between different factors iv. Important information from the raw data is lost as it uses the rank-based method.

## Challenges with these common methods

### Infection prevalence

Simple mean prevalence estimates are affected by a small number of very high or very low (zero) values in treatment groups, and errors are higher if replicates show high variability (usually due to uncontrolled experimental variables). Prevalence values are commonly close to zero, especially in field-collected mosquitoes. In addition, the noted variation in prevalence in replicates due to uncontrolled (but measurable) experimental variables, such as gametocyte density or mosquito size, is not incorporated into simple mean prevalence estimates.

### Infection intensity

Typically, within an experiment, there are usually a few mosquitoes with very high oocyst numbers and some with zero or very low numbers, even within relatively homogeneous lab-reared mosquitoes fed on the same infectious blood meal. Oocyst numbers within mosquitoes typically exhibit overdispersion (Sinden et al., 2007; Vaughan, 2007; Churcher et al., 2012) and are not normally distributed, i.e., not a symmetrically distributed dataset with a bell shape, and many values do not cluster in the central portion when plotted on the graph (reviewed in Bhandari, 2023). Using mean values to estimate oocyst numbers will be highly influenced by extreme values and skewed distributions, and will usually overestimate the true value. Median values are often a better summary statistic, but for oocyst distributions, the median value will be zero if the prevalence is less than 50% (which it frequently is). Oocyst intensity data often more closely approximate a negative binomial (NB), often with zero-inflation (Miura et al., 2019; Akorli et al., 2022). This type of data cannot usually be transformed by standard methods e.g. logs, making a mean oocyst number an inappropriate measure.

How then should mosquito infection data with these types of distribution and with different experimental variables or treatments be analysed?

## Use of generalized linear models

A generalized linear model (GLM) is a statistical method useful for analysing non-normal datasets. It models or analyses datasets with only fixed effects, which are explanatory variables (predictors or inputs/independent variables), such as different drug treatments or number of gametocytes in bloodmeal, with a constant effect across different individuals or groups. A GLM mainly consists of a linear predictor, a link function and an error distribution. A linear predictor is the part of the model where all the explanatory variables are combined to give or predict the result. A link function connects a linear predictor to what the model wants to predict, for example a log-link function is common for count data. The error distribution, also known as family, defines the distribution on which the model is based, for example Poisson or NB (McCullagh and Nelder, 1989). The model uses a linear formula to combine explanatory variables and predict outcomes using link functions and exponential family distributions, such as normal, Poisson or binomial distributions (Bolker et al., 2009). The type of family distribution to be chosen when using GLM depends on the nature of the dataset (e.g., binary, continuous or count data). This makes GLM flexible and valuable for different types of datasets.

## Advantages of GLMs

A GLM analysis eliminates the challenge of data transformation with non-normal data and accommodates different types of datasets, as it allows response variables to have different distributions. A GLM approach is very flexible in analysing the relationship between variables using a link function that handles non-linear relationships, and predicts results that are simple to interpret.

A further extension of the GLM is the Generalised Linear Mixed model (GLMM), which can model datasets with random effects. Random effects are unmeasurable variables that differ among repeated measurements, such as differences between biological replicates due to unknown factors. GLMMs are better statistical tools than GLMs for analysing non-normal data involving random effects, or when random effects are the focus of the analyses. Defining model predictors in GLMM either as fixed or random effects is essential in modelling; the important criteria to consider before using GLMM and fitting random variables have been discussed previously (Harrison et al., 2018). The conditions of when to include a random effect in a model, and the challenges of using GLMM for non-experts, have also been discussed extensively (Bolker et al., 2009). One condition to fulfil before using random effects in a model is that at least 5 ‘levels or group’ (e.g., number of biological replicates or number of mosquito cages) must be achieved in the dataset to be able to estimate variance (Gelman and Hill, 2006; Kéry and Royle, 2015; Harrison et al., 2018; Gomes, 2022). As a result of this pitfall, non-experts could use GLMM inappropriately (Bolker et al., 2009), for example when there are fewer than five replicates of each experimental treatment.

A GLMM can be the best model for analysing mosquito infection data involving different parasite drug treatments, many biological replicates, multiple feeders/mosquito cages and mosquito populations, to account for random effects/or variability (Churcher et al., 2012). However, for most SMFA experiments, the number of biological replicates rarely exceeds 5, which could rule out using GLMM to analyse such a dataset where biological replicate is a potential random effect.

The following examples illustrate the use of a GLM approach to investigate the impact of transmission blocking drugs on the prevalence and infection of *P. falciparum* in mosquitoes in an SMFA.

### Example 1: GLM analysis of infection prevalence

**Data**: The data used for illustration (Table 3) are adapted from an examination of the transmission-blocking properties of antimalarial drugs. Transmission stages (gametocytes) of *P. falciparum* were grown *in vitro*, exposed to different drugs during development, and then used in experimental infections of *Anopheles* mosquitoes in an SMFA. The prevalence and intensity of the resulting oocyst infection was determined by mosquito dissection 10 days after the infectious blood meal, and recording the presence and number of oocysts.

**Table 3.** Raw data of prevalence and gametocyte density for three biological replicates

	Replicate A		Replicate B		Replicate C		
Drug treatment	Prevalence	GC density (per 10 000 rbc)	Prevalence	GC density (per 10 000 rbc)	Prevalence	GC density (per 10 000 rbc)	Mean prevalence
Control	87%	17	85%	20	88%	11	86%
Drug_A	90%	16	88%	18	96%	14	91%
Drug_B	82%	12	77%	11	67%	12	75%
Drug_C	14%	7	90%	7	65%	12	57%
Drug_D	0%	8	4%	6	4%	4	3%
Drug_E	0%	3	0%	5	0%	2	0%

Gametocytes were treated with five different drugs (A–E), and the control group was untreated, and were then used to infect *Anopheles* mosquitoes by SMFA. Three biological replicates were performed. Values are prevalence of infection in mosquitoes (*n* = 30 mosquitoes examined for infection for each drug). Gametocyte (GC) density was evaluated by examination of at least 10 000 red blood cells (rbc) in a thin Giemsa-stained smear.

**Analysis**: To illustrate the statistical methods recommended here, the prevalence of infection was compared by the commonly used one-tailed Student’s *t*-test, and by standard logistic regression GLM, and logistic regression GLM with Firth’s bias reduction (using the R package logistf (Heinze et al., 2023)) (Table 4). Both GLM models incorporated the variables of gametocyte density in the blood meal and replicate as fixed variables. The significance of the experimental variables was tested using a backward elimination method (Burnham and Anderson, 1998). This begins with a maximal model containing all experimental variables, and is then simplified by stepwise elimination of non-significant factors until the final model, containing only significant effects, is reached.

**Table 4.** Predicted prevalences obtained from the two GLM models (with 95% confidence intervals), with the mean prevalence (and standard error) for comparison

		Predicted prevalence % (95% CI)		Statistical tests for comparison with control		
Drug treatment	Mean prevalence (s.e.)	GLM	Logistf	*P* value *t*-test	*P* value GLM	*P* value logistf
Control	86.4 (0.98)	87.2 (77.0−93.3)	86.5 (76.3−92.8)	n/a	n/a	n/a
Drug_A	91.3 (2.40)	92.1 (83.3−96.5)	91.4 (82.5−96.0)	0.916	0.340	0.348
Drug_B	75.3 (4.58)	75.0 (63.0−84.0)	74.4 (62.6−83.4)	0.064	0.074	0.074
Drug_C	56.5 (22.62)	58.0 (45.8−69.3)	57.8 (45.8−69.0)	0.158	**0.0003**	**0.0001**
Drug_D	2.8 (1.24)	2.8 (0.7−10.6)	3.5 (1.0−11.5)	**6.57 × 10^−7^**	1.62 × 10**^−11^**	**0.0000**
Drug_E	0.0 (0)	8.2 × 10^−7^ (2.2 × 10^−16^)	0.8 (0.5−10.7)	**6.47× 10^−5^**	0.98	**0.0000**

*P* values represent the significance of the difference in prevalence observed between the control and each drug-treated group, as produced by the different analysis methods (Student’s *t*-test, GLM, GLM/logistf). *P* values <0.05 are highlighted in bold.

**Figure 1.**
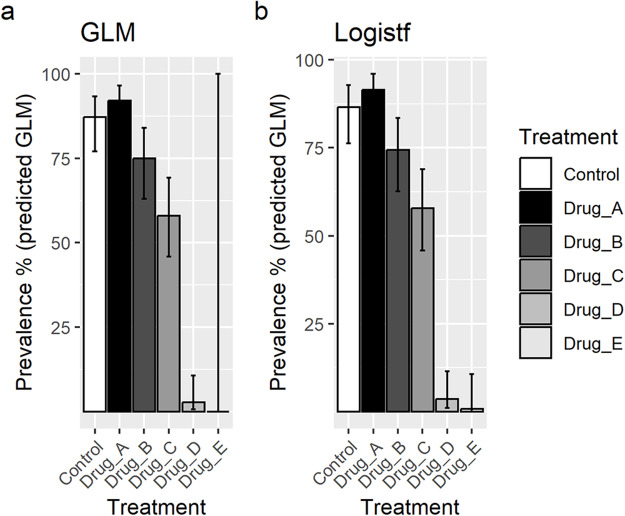
Graphical representation of the predicted prevalence with 95% confidence intervals obtained by the two GLM methods: (A) standard logistic regression by GLM (B) logistic regression with Firth’s bias reduction (using the r package logistf).

Comparisons of each drug compared to the untreated control are also shown in Table 4. Predictions of prevalence were obtained for the 2 GLM methods (see supplementary material for R codes and packages used) for comparison with the mean prevalence over all 3 biological replicates (Table 4 and Figure 1).

**Results**: The simple comparison of mean prevalence using Student’s *t*-test identified significant differences of infection prevalence in mosquitoes between the untreated control and gametocytes treated with drug D or drug E, but with no significant effect of drugs A, B or C (Table 4). The GLM approaches allowed the inclusion of additional variables such as gametocyte density, which can be seen to vary between treatment groups and between replicates (Table 3), as well as differences in overall prevalence in the controls between replicates, which includes non-measured sources of variation. The standard logistic regression by GLM revealed a significantly lower infection prevalence in gametocytes treated with drug C compared to controls (Table 4), but this standard GLM approach was unsatisfactory in the group treated with drug E, where transmission was completely blocked. The use of logistic regression with Firth’s bias reduction, using the R package logistf, overcame this issue, and allowed comparisons of prevalence in each group from the model.

The use of GLM/logistf thus improved the estimate of the impact of the drug on transmission compared to the Student’s *t*-test analysis, revealing a significant difference between the untreated control and three of the five drugs tested, whereas the *t*-test analysis found only 2 drugs to have significant transmission-blocking effects.

### Example 2: GLM estimation of the oocyst intensity data

**Figure 2.**
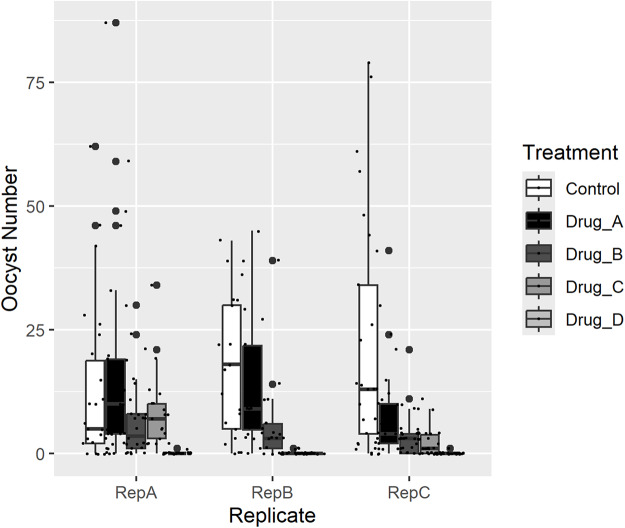
Raw data of oocyst numbers for three biological replicates. Each individual dot represents a single mosquito.

**Data**: The experiment described in Example 1 included counts of oocyst numbers for individual mosquitoes, determined by dissection 10 days after the infectious blood meal. The group treated with drug E was removed from the analysis because there was zero prevalence, and therefore no oocyst numbers for which a distribution can be modelled. A summary of the data is shown in Table 5 and Figure 2.

**Table 5.** Mean and median oocyst numbers for three biological replicates of the infections described in Example 1.

	Oocyst numbers per mosquito					
	Replicate A		Replicate B		Replicate C	
Drug treatment	Mean	Median	Mean	Median	Mean	Median
Control	17.82	18.00	12.69	5.00	22.96	13.00
Drug_A	14.05	9.00	17.48	10.00	7.40	4.00
Drug_B	6.29	3.00	6.42	3.50	3.29	3.00
Drug_C	0.14	0.00	8.62	7.00	2.12	1.00
Drug_D	0.00	0.00	0.04	0.00	0.04	0.00

Gametocytes were treated with four different drugs (A–D), and the control group was untreated. Values are oocyst intensity in mosquitoes (*n* = 30 mosquitoes examined for oocyst intensity for each drug).

**Figure 3.**
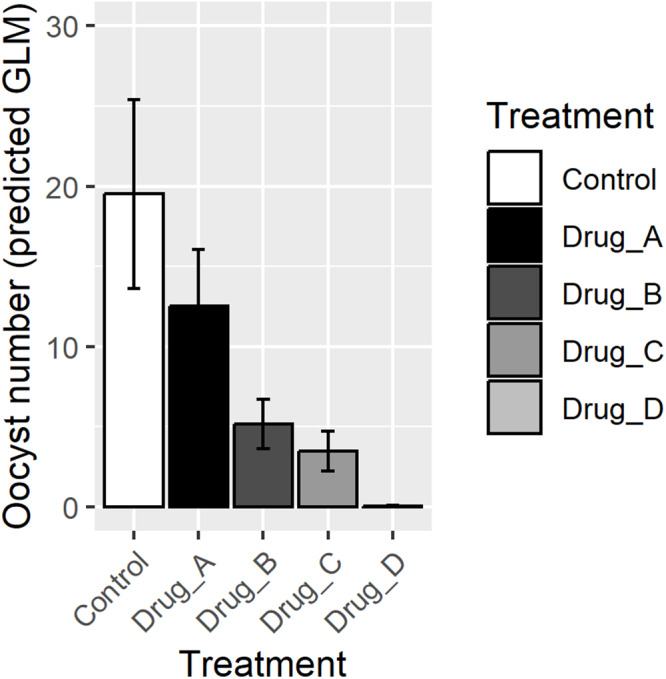
Graphical representation of the predicted intensity with 95% confidence intervals obtained by the GLM approach (here, a zero-inflated negative binomial model).

**Analysis**: The analysis was performed using the methods commonly used in the published literature: comparisons of median oocyst numbers using non-parametric tests (Kruskal–Wallis), and of mean oocyst intensity by Student’s *t*-test/ANOVA (despite its likely unsuitability for the data distributions) (Table 6). The results were then compared with those obtained by the GLM approach (Table 7). For the GLM models, different drugs (A–D), gametocyte density and biological replicate were used as fixed variables, and the distributions tested were Poisson, quasi-Poisson, NB, zero-inflated negative binomial (ZINB) and hurdle NB. The best-fit GLM for the data was assessed using the lowest Akaike Information Criterion and the best prediction of the zero counts in the data set (see supplementary material for the R codes used). Predictions of oocyst intensity for each drug were obtained for the GLM method (Table 7 and Figure 3). Comparisons of each drug treatment compared to the untreated control are shown in Table 7.

**Table 6.** Significance tests for analysis of oocyst numbers (infection intensity) by two methods commonly used in the published literature (Kruskal–Wallis test and ANOVA)

	Kruskal–Wallis				
Variable and comparisons	Rep A	Rep B	Rep C	Pooled replicates	ANOVA
Treatment	**5.7 × 10^−12^**	**2.7 × 10^−9^**	**8.6 × 10^−12^**	**2.2 × 10^−16^**	**2 × 10^−16^**
Control vs Drug A	0.464	0.367	**0.026**	0.239	0.365
Control vs Drug B	**0.02**	0.342	**0.0005**	**2.9 × 10^−5^**	**0.000**
Control vs Drug C	**2.1 × 10^−6^**	0.957	**7.4 × 10^−5^**	**1.8 × 10^−8^**	**0.000**
Control vs Drug D	**3.7 × 10^−6^**	**1.2 × 10^−7^**	**7.1 × 10^−8^**	**<2 × 10^−16^**	**0.000**

The values are *P* values for Kruskal–Wallis and ANOVA tests, the latter analysed on pooled data and then individually by replicate. *P* values <0.05 are highlighted in bold.

**Table 7.** Predicted infection intensity obtained from the best-fit GLM model (with 95% confidence intervals), and significance of the difference in intensity observed between the control and each drug-treated group

Treatment group	Predicted oocyst number from GLM (95% CI)	*P* value (GLM) comparison with control
Control	19.5 (13.6–25.4)	–
Drug_A	12.5 (9.0–16.0)	**0.030**
Drug_B	5.2 (3.6–6.7)	**5.1 × 10^−10^**
Drug_C	3.5 (2.2–4.7)	**2.3 × 10^−8^**
Drug_D	0.0 (0.0−0.1)	**1.1 × 10^−14^**

*P* values <0.05 are highlighted in bold.

**Results**: A non-parametric test (Kruskal–Wallis test) identifies significant differences in oocyst numbers between the untreated control and infections in which gametocytes were treated with drugs B, C or D, but this approach does not permit the inclusion of additional variables, including replicate. Although the data violate the assumptions for analysis by parametric tests such as ANOVA because of the non-normal distribution and high numbers of zero values in some groups, the results are shown for completeness (Table 6). The GLM approach (Table 7) allows modelling to fit several different distributions and the selection of the most appropriate for the given data set, as well as allowing inclusion of additional variables. For these data, the best model (selected on the basis on the log likelihood and the prediction of zero events; see supplementary material) was the ZINB model including fixed effects of treatment and replicate (gametocyte density was not a significant explanatory factor, *P* = 0.064). The application of GLM improved the estimate of the drug effect compared to the non-parametric method (Kruskal–Wallis), revealing a significant difference between the untreated control and all of the drugs tested.

## Insights from the analyses

The GLM approach illustrated here examines infection prevalence and oocyst intensity in mosquitoes with real data taken from an examination of the transmission-blocking properties of different drugs. As the number of biological replicates was 3, replicate was not included as a random effect but as a fixed effect in the GLM. To account for additional experimental variables and determine their effect on the prevalence of mosquito infection and oocyst intensity, the infection prevalence was analysed by fitting it to a binomial distribution (probability of infection) using logistic regression, while oocyst intensity raw data were fitted by ZINB and the results were compared with those obtained by the commonly seen analysis methods.

For prevalence of infection, the simplest Student’s *t*-test result did not identify a significant impact of drug C on transmission, which was however detected using the GLM/logistf approach (Table 4). For impact on oocyst numbers (Table 6), the simplest analysis using a Kruskal–Wallis analysis identified a significant difference in oocyst intensity following treatment with drugs A and B, but this effect was not seen in all three replicates, leading to the conclusion that drug A did not affect oocyst numbers. The GLM approach allowed the inclusion of replicate, and revealed a significant decrease in oocyst numbers between the untreated control and all the drugs tested (A, B, C and D). The use of a GLM approach thus improved the estimate of the impact of the intervention, and identified **all** four drugs tested as effective in lowering transmission oocyst intensity compared to the simple Kruskal–Wallis analysis.

Statistical interactions between different explanatory variables and multiple comparisons, which were not performed for this example dataset for simplicity, could also provide more insight. We focused on the main effects to give an example of the advantages of GLM over common, simple tests.

## Recommendations

A well-chosen GLM (or where appropriate, GLMM) approach can handle the problematic issues identified with non-normal distributions and overdispersion; additional variables such as gametocyte density and biological replicates can be included, and their impact on mosquito infections can be assessed.

Infection prevalence can be analysed using a logistic regression GLM, incorporating additional measurable variables (such as gametocyte density or mosquito size) where they have a statistically significant effect. However, mosquito infection prevalence can be close to zero, or zero in some groups, especially when examining effective transmission-blocking interventions or infections from MFA. This results in a quasi-complete separation of data if standard logistic regression (basic GLM in R) is used to fit the data, and the software may report an error or warning, or the standard errors for the model will be extremely large (as seen in Figure 1A for drug E). Quasi-complete separation of data occurs when a predictor gives a perfect prediction of a response variable to a certain degree or for most values of the predictors, but not all values, for example, when treatment with a drug gives zero or close to zero infection (Albert and Anderson, 1984; Advanced Research Computing, 2021). This problem can be addressed by analysing the data using Firth’s bias reduction method (Firth, 1993) for the GLM, which can be accomplished with the R package ‘logistf’ (Heinze et al., 2023). Logistf fits a logistic regression model with Firth’s bias reduction method, a penalized maximum likelihood method which reduces the bias and improves convergence in the presence of data with an excess or high number of zeros (Heinze et al., 2023). This provides an ideal solution to the problem of separation in logistic regression (Heinze and Schemper, 2002).

For analysis of infection intensity, GLMs can fit a number of different distributions that more closely match the data. The user defines the distribution to be used, and the fit of the data to the chosen distribution can be assessed and the best distribution that reflects the data selected.

Previous studies have suggested that a NB regression model is usually better at explaining the distribution of *Plasmodium* oocysts in the mosquito than a normal or Poisson distribution (Medley et al., 1993; Billingsley et al., 1994). A NB model is a statistical model of success/failure outcomes that fits over-dispersed count data when the variance exceeds the mean (Cummings and Hardin, 2019). A ZINB, which accounts for excess zeros in a dataset in two ways, i.e. ‘true zeros’ and ‘excess zeros’ (Cummings and Hardin, 2019), can often give an even better estimation than the NB model, as has been shown for both *P. berghei* and *P. falciparum* experimental infections (Churcher et al., 2012; Swihart et al., 2018).

Therefore, a better alternative to using mean or median estimates of oocyst intensity is the use of GLM or GLMM to fit the oocyst numbers (data) to alternative distributions to which they are better matched, e.g. NB or ZINB, which allows better estimates of infection intensity, as well as comparison between groups. A GLM approach also allows external variables (such as gametocyte density) to be included in the oocyst number estimates. We have applied this approach recently to model *P. falciparum* prevalence and oocyst intensity in mosquitoes under different gametocyte growth conditions (Pradhan et al., 2024). Other authors have used a similar approach: a ZINB model that includes random effects to analyse mosquito oocyst intensity (Churcher et al., 2012; Miura et al., 2013; McGuire et al., 2017; Swihart et al., 2018; Chan et al., 2019; Lee et al., 2019; Balam et al., 2025; Naghizadeh et al., 2025).

## Conclusions

The advantages of using GLM analyses, with appropriate distributions, to analyse the typically non-normal data obtained from human malaria parasite infections in *Anopheles* mosquitoes are presented here. In the examples given of analysis of both prevalence and intensity of infection, the GLM method identified more significant transmission blocking effects among the drugs tested than simpler analyses that are frequently used in publications.

By providing evidence of the added benefits of these methods, as well as the R codes used, we hope to persuade researchers to apply these analytical techniques. We recommend that researchers use logistic regression GLM, (or GLMM if appropriate) as an alternative to the use of mean/median estimates for the estimation and interpretation of mosquito infection prevalence with *P. falciparum*, and other parasitic infections with zero or close to zero infection prevalence. For infection intensity, the fitting of GLM (or GLMM) with different distributions, and then selecting the best model for the data, is a little more complex, but gives a more robust and informative analysis, avoiding the pitfalls and disadvantages of parametric (ANOVA) and simple non-parametric (Kruskal–Wallis) methods.

While the focus was on the use of these statistical approaches for the evaluation of mosquito infection data, such non-normal distributions are common in many parasitic diseases, where a minority of individuals harbour a majority of parasites, and the majority harbour low or zero numbers. These analytical methods can equally be utilized by researchers working on other parasitic control interventions, to make best use of their data.

## Supporting information

Ubiaru and Ranford-Cartwright supplementary material Ubiaru and Ranford-Cartwright supplementary material

## Supplementary material

The supplementary material for this article can be found at https://doi.org/10.1017/S0031182025100541.

## References

[ref1] Advanced Research Computing. (2021). *What is complete or quasi-complete separation in logistic/probit regression and how do we deal with them*. Available: https://stats.oarc.ucla.edu/other/mult-pkg/faq/general/faqwhat-is-complete-or-quasi-complete-separation-in-logisticprobit-regression-and-how-do-we-deal-with-them/#:∼:text=In%20this%20example%2C%20Y%20is%20the,other%20words%2C%20Y%20separates%20X1%20perfectly.&text=In%20this%20example%2C%20Y,Y%20separates%20X1%20perfectly.&text=example%2C%20Y%20is%20the,other%20words%2C%20Y%20separates (accessed 5 January 2023).

[ref2] Akorli EA, Ubiaru PC, Pradhan S, Akorli J and Ranford-Cartwright L (2022) Bio-products from *Serratia marcescens* isolated from Ghanaian *Anopheles gambiae* reduce *Plasmodium falciparum* burden in vector mosquitoes. *Frontiers in Tropical Diseases* 3, 979615. 36742111 10.3389/fitd.2022.979615 PMC7614139

[ref3] Albert A and Anderson JA (1984) On the existence of maximum likelihood estimates in logistic regression models. *Biometrika* 71, 1–10.

[ref4] Balam S, Miura K, Ayadi I, Konaté D, Incandela NC, Agnolon V, Guindo MA, Diakité S, Olugbile S, Nebie I, Herrera SM, Long C, Kajava AV, Diakité M, Corradin G, Herrera S and Herrera MA (2025) Cross-reactivity of rPvs48/45, a recombinant *Plasmodium vivax* protein, with plasma from *Plasmodium falciparum* endemic areas of Africa. *PLoS One* 20, e0302605. 40100850 10.1371/journal.pone.0302605 PMC11918314

[ref5] Bhandari P (2023) Normal Distribution: Examples, Formulas, & Uses. *Scribbr*, Retrieved September 10, 2024, from https://www.scribbr.com/statistics/normal-distribution/

[ref6] Billingsley PF, Medley GF, Charlwood D and Sinden RE (1994) Relationship Between Prevalence and Intensity of *Plasmodium falciparum* Infection in Natural Populations of *Anopheles* Mosquitoes. *The American Journal of Tropical Medicine and Hygiene* 51, 260–270. 7943543 10.4269/ajtmh.1994.51.260

[ref7] Bolker BM, Brooks ME, Clark CJ, Geange SW, Poulsen JR, Stevens MH and White JS (2009) Generalized linear mixed models: A practical guide for ecology and evolution. *Trends in Ecology and Evolution* 24, 127–135. 19185386 10.1016/j.tree.2008.10.008

[ref8] Bousema T, Dinglasan RR, Morlais I, Gouagna LC, Van Warmerdam T, Awono-Ambene PH, Bonnet S, Diallo M, Coulibaly M, Tchuinkam T, Mulder B, Targett G, Drakeley C, Sutherland C, Robert V, Doumbo O, Touré Y, Graves PM, Roeffen W, Sauerwein R, Birkett A, Locke E, Morin M, Wu Y and Churcher TS (2012) Mosquito feeding assays to determine the infectiousness of naturally infected *Plasmodium falciparum* gametocyte carriers. *PLoS One* 7, e42821. 22936993 10.1371/journal.pone.0042821 PMC3425579

[ref9] Burnham KP and Anderson DR (1998) *Model Selection and Inference: A Practical Information-Theoretical Approach*. New York: Springer-Verlag.

[ref10] Chan JA, Wetzel D, Reiling L, Miura K, Drew DR, Gilson PR, Anderson DA, Richards JS, Long CA, Suckow M, Jenzelewski V, Tsuboi T, Boyle MJ, Piontek M and Beeson JG (2019) Malaria vaccine candidates displayed on novel virus-like particles are immunogenic and induce transmission-blocking activity. *PLoS One* 14, e0221733. 31504038 10.1371/journal.pone.0221733 PMC6736250

[ref11] Churcher TS, Blagborough AM, Delves M, Ramakrishnan C, Kapulu MC, Williams AR, Biswas S, Da DF, Cohuet A and Sinden RE (2012) Measuring the blockade of malaria transmission–an analysis of the Standard Membrane Feeding Assay. *International Journal for Parasitology* 42, 1037–1044. 23023048 10.1016/j.ijpara.2012.09.002

[ref12] Cummings TH and Hardin JW (2019) Modeling count data with marginalized zero-inflated distributions. *The Stata Journal* 19, 499–509.

[ref13] Firth D (1993) Bias reduction of maximum likelihood estimates. *Biometrika* 80, 27–38.

[ref14] Gelman A and Hill J (2006) *Data Analysis Using Regression and Multilevel/hierarchical Models*. Cambridge: Cambridge University Press.

[ref15] Gomes DGE (2022) Should I use fixed effects or random effects when I have fewer than five levels of a grouping factor in a mixed-effects model? *PeerJ* 10, e12794. 35116198 10.7717/peerj.12794 PMC8784019

[ref16] Harrison XA, Donaldson L, Correa-Cano ME, Evans J, Fisher DN, Goodwin CED, Robinson BS, Hodgson DJ and Inger R (2018) A brief introduction to mixed effects modelling and multi-model inference in ecology. *PeerJ* 6, e4794. 29844961 10.7717/peerj.4794 PMC5970551

[ref17] Heinze G, Ploner M, Jiricka L and Steiner G (2023) logistf: Firth’s Bias-Reduced Logistic Regression https://CRAN.R-project.org/package=logistf. *R package version 1.26.0*.

[ref18] Heinze G and Schemper M (2002) A solution to the problem of separation in logistic regression. *Statistics in Medicine* 21, 2409–2419. 12210625 10.1002/sim.1047

[ref19] Ippolito MM, Moser KA, Kabuya JB, Cunningham C and Juliano JJ (2021) Antimalarial Drug Resistance and Implications for the WHO Global Technical Strategy. *Current Epidemiology Reports* 8, 46–62. 33747712 10.1007/s40471-021-00266-5 PMC7955901

[ref20] Kefi M, Cardoso-Jaime V, Saab SA and Dimopoulos G (2024) Curing mosquitoes with genetic approaches for malaria control. *Trends in Parasitology* 40, 487–499. 38760256 10.1016/j.pt.2024.04.010

[ref21] Kéry M and Royle JA (2015) *Applied Hierarchical Modeling in Ecology: analysis of Distribution, Abundance and Species Richness in R and BUGS: prelude and Static Models*. Waltham: Academic Press.

[ref22] Lee SM, Wu Y, Hickey JM, Miura K, Whitaker N, Joshi SB, Volkin DB, Richter King C and Plieskatt J (2019) The Pfs230 N-terminal fragment, Pfs230D1+: Expression and characterization of a potential malaria transmission-blocking vaccine candidate. *Malaria Journal* 18, 356. 31703583 10.1186/s12936-019-2989-2 PMC6839146

[ref23] Lyimo EO and Koella JC (1992) Relationship between body size of adult *Anopheles gambiae s.l*. and infection with the malaria parasite *Plasmodium falciparum*. *Parasitology* 104(Pt 2), 233–237. 1594289 10.1017/s0031182000061667

[ref24] McCullagh P and Nelder JA (1989) *Generalized Linear Models*. London; New York: Chapman and Hall.

[ref25] McGuire KA, Miura K, Wiethoff CM and Williamson KC (2017) New adenovirus-based vaccine vectors targeting Pfs25 elicit antibodies that inhibit *Plasmodium falciparum* transmission. *Malaria Journal* 16, 254. 28619071 10.1186/s12936-017-1896-7 PMC5471885

[ref26] Medley GF, Sinden RE, Fleck S, Billingsley PF, Tirawanchai N and Rodriguez MH (1993) Heterogeneity in patterns of malarial oocyst infections in the mosquito vector. *Parasitology* 106(Pt 5), 441–449. 8341579 10.1017/s0031182000076721

[ref27] Miura K, Deng B, Tullo G, Diouf A, Moretz SE, Locke E, Morin M, Fay MP and Long CA (2013) Qualification of standard membrane-feeding assay with *Plasmodium falciparum* malaria and potential improvements for future assays. *PLoS One* 8, e57909. 23483940 10.1371/journal.pone.0057909 PMC3590281

[ref28] Miura K, Swihart BJ, Deng B, Zhou L, Pham TP, Diouf A, Burton T, Fay MP and Long CA (2016) Transmission-blocking activity is determined by transmission-reducing activity and number of control oocysts in *Plasmodium falciparum* standard membrane-feeding assay. *Vaccine* 34, 4145–4151. 27372156 10.1016/j.vaccine.2016.06.066 PMC4958521

[ref29] Miura K, Swihart BJ, Deng B, Zhou L, Pham TP, Diouf A, Fay MP and Long CA (2019) Strong concordance between percent inhibition in oocyst and sporozoite intensities in a *Plasmodium falciparum* standard membrane-feeding assay. *Parasites and Vectors* 12, 206. 31060594 10.1186/s13071-019-3470-3 PMC6501457

[ref30] Naghizadeh M, Miura K, Addo Ofori E, Long C, Sagara I, Tiono AB, Plieskatt J and Theisen M (2025) Magnitude and durability of ProC6C-AlOH/Matrix-M(tm) vaccine-induced malaria transmission-blocking antibodies in Burkinabe adults from a Phase 1 randomized trial. *Human Vaccines and Immunotherapeutics* 21, 2488075. 40208198 10.1080/21645515.2025.2488075 PMC11988263

[ref31] Ohm JR, Baldini F, Barreaux P, Lefevre T, Lynch PA, Suh E, Whitehead SA and Thomas MB (2018) Rethinking the extrinsic incubation period of malaria parasites. *Parasites and Vectors* 11, 178. 29530073 10.1186/s13071-018-2761-4 PMC5848458

[ref32] Paterson S and Lello J (2003) Mixed models: Getting the best use of parasitological data. *Trends in Parasitology* 19, 370–375. 12901939 10.1016/s1471-4922(03)00149-1

[ref33] Ponnudurai T, Lensen AHW, Vangemert GJA, Bensink MPE, Bolmer M and Meuwissen JHET (1989) Infectivity of Cultured *Plasmodium-Falciparum* Gametocytes to Mosquitos. *Parasitology* 98, 165–173. 2668861 10.1017/s0031182000062065

[ref34] Ponnudurai T, Van Gemert GJ, Bensink T, Lensen AHW and Meuwissen JHET (1987) Transmission blockade of *Plasmodium falciparum*: Its variability with gametocyte numbers and concentration of antibody. *Transactions of the Royal Society of Tropical Medicine and Hygiene* 81, 491–493. 3318022 10.1016/0035-9203(87)90172-6

[ref35] Pradhan S, Ubiaru PC and Ranford-Cartwright L (2024) Simple supplementation of serum-free medium produces gametocytes of *Plasmodium falciparum* that transmit to mosquitoes. *Malaria Journal* 23, 275. 39256807 10.1186/s12936-024-05094-8 PMC11389287

[ref36] Shaw WR, Holmdahl IE, Itoe MA, Werling K, Marquette M, Paton DG, Singh N, Buckee CO, Childs LM and Catteruccia F (2020) Multiple blood feeding in mosquitoes shortens the *Plasmodium falciparum* incubation period and increases malaria transmission potential. *PLoS Pathogens* 16, e1009131. 33382824 10.1371/journal.ppat.1009131 PMC7774842

[ref37] Sinden RE, Dawes EJ, Alavi Y, Waldock J, Finney O, Mendoza J, Butcher GA, Andrews L, Hill AV, Gilbert SC and Basáñez MG (2007) Progression of Plasmodium berghei through Anopheles stephensi is density-dependent. *PLoS Pathogens* 3, e195. 18166078 10.1371/journal.ppat.0030195 PMC2156095

[ref38] Suh PF, Elanga-Ndille E, Tchouakui M, Sandeu MM, Tagne D, Wondji C and Ndo C (2023) Impact of insecticide resistance on malaria vector competence: A literature review. *Malaria Journal* 22, 19. 36650503 10.1186/s12936-023-04444-2 PMC9847052

[ref39] Swihart BJ, Fay MP and Miura K (2018) Statistical methods for standard membrane-feeding assays to measure transmission blocking or reducing activity in malaria. *Journal of the American Statistical Association* 113, 534–545. 31007315 10.1080/01621459.2017.1356313 PMC6469714

[ref40] Vaughan JA (2007) Population dynamics of Plasmodium sporogony. *Trends in Parasitology* 23, 63–70. 17188574 10.1016/j.pt.2006.12.009

[ref41] Vaughan JA, Noden BH and Beier JC (1992) Population dynamics of *Plasmodium falciparum* sporogony in laboratory-infected *Anopheles gambiae*. *The Journal of Parasitology* 78, 716–724. 1635032

[ref42] World Health Organisation (2024). *World Malaria Report 2024*. Available: https://www.who.int/teams/global-malaria-programme/reports/world-malaria-report-2024 (accessed 8 April 2025).

[ref43] Wu Y, Sinden RE, Churcher TS, Tsuboi T and Yusibov V (2015) Development of malaria transmission-blocking vaccines: From concept to product. *Advances in Parasitology* 89, 109–152. 26003037 10.1016/bs.apar.2015.04.001

[ref44] Yu S, Wang J, Luo X, Zheng H, Wang L, Yang X and Wang Y (2022) Transmission-blocking strategies against malaria parasites during their mosquito stages. *Frontiers in Cellular & Infection Microbiology* 12, 820650. 35252033 10.3389/fcimb.2022.820650 PMC8889032

