# Supplementary material for: When a mean can be meaningless: evaluating mosquito infections with Plasmodium parasites
Source: Parasitology. 2025 Jul 14;152(9):881–9. doi: 10.1017/S0031182025100541 (PMC12644941; doi:10.1017/S0031182025100541)
Supplement: Ubiaru and Ranford-Cartwright supplementary material [file S0031182025100541sup001.docx]

# Supplementary material

## R codes and packages for analyses

#load R packages needed for analysis

library(ggplot2) #R package for general plotting

library(lme4) # R package for logistic GLM

library(effects) # R package for predictions from GLMs

library(ggeffects) # R package for predictions from GLMs

library(MASS) # R package for negbin GLM

library(pscl) # R package for ZINB and hurdle models

# dataset is imported from excel file and named "data1". Explanatory variables are different drugs (treatment), gametocyte density in blood meal (bldml_gc) and replicate (feedno). Response variables are infection status (0/1; infected) and infection intensity (oocyst count; (no_oocysts).

# logistic glm for data1 -----------------------------------------------------

# set some explanatory variables as factors

data1$feedno <- as.factor(data1$feedno)

data1$treatment <- as.factor(data1$treatment)

#glm full model with all variables

mod1_db4<-glm(infected~ treatment+bldml_gc+feedno, family=binomial, data = data1)

summary(mod1_db4)

# backward elimination of full model : remove non-significant terms - compare full model to one without feedno

mod1_db4b<-glm(infected~ treatment+bldml_gc, family=binomial, data = data1)

summary(mod1_db4b)

anova(mod1_db4, mod1_db4b)

# backward elimination of full model : remove non-significant terms - compare without bldml_GC

mod1_db4c<-glm(infected~ treatment+feedno, family=binomial, data = data1)

summary(mod1_db4c)

anova(mod1_db4, mod1_db4c)

# backward elimination of full model : remove non-significant terms - compare without both

mod1_db4d<-glm(infected~ treatment, family=binomial, data = data1)

summary(mod1_db4d)

anova(mod1_db4, mod1_db4d)

#Conclusion on best model: here mod1_db4c is best model

#predictions of prevalence from bestfit model using R package effects

effects_mod1_db4c<-allEffects(mod1_db4c)

plot(effects_mod1_db4c, lines=list(lty=0))

df_eff_mod1_db4c<-as.data.frame(effects_mod1_db4c)

df_eff_mod1_db4c

#draw better graph of predicted results using R package ggplot

mod1_db4c_Fig<-ggplot(df_eff_mod1_db4c$treatment, aes(y=fit*100, x=treatment, fill=treatment)) +

geom_bar(color="black", stat="identity", position=position_dodge()) +

geom_errorbar(aes(ymin=lower*100, ymax=upper*100), width=0.2) +

ylab("Prevalence % (predicted GLM)") +

xlab("Treatment") +

ggtitle("GLM")+

ylim(0, 120)

mod1_db4c_Fig + scale_y_continuous(breaks=c(25,50, 75, 100))

# prediction for drug E has extended error bars - needs package logistf (as all zero in this group)

# Run logistf for db4 -----------------------------------------------------------------

logf1_db4<-logistf(infected~ treatment+feedno+bldml_gc,firth = TRUE, data = data1)

summary(logf1_db4)

# backward elimination of full model : remove non-significant terms - compare without bldml_GC

logf2_db4<-logistf(infected~ treatment+feedno,firth = TRUE, data = data1)

summary(logf2_db4)

anova(logf1_db4, logf2_db4)

# backward elimination of full model : remove non-significant terms - compare without feedno

logf3_db4<-logistf(infected~ treatment+bldml_gc,firth = TRUE, data = data1)

summary(logf3_db4)

anova(logf1_db4, logf3_db4)

# backward elimination of full model : remove non-significant terms - compare without bldml_GC and feedno

logf4_db4<-logistf(infected~ treatment,firth = TRUE, data = data1)

summary(logf4_db4)

anova(logf1_db4, logf4_db4)

anova(logf2_db4, logf4_db4)

#select best model: here logf2_db4 which includes treatment and feedno

#obtain predictions of model using R package effects

effectsmodel_Logf2_db4<-allEffects(logf2_db4)

plot(effectsmodel_Logf2_db4, lines=list(lty=0))

effectsmodel_Logf2_db4

Logf2_db4_eff<-as.data.frame(effectsmodel_Logf2_db4)

Logf2_db4_eff

#draw better graph of predicted results using R package ggplot

logf2_db4_Fig<-ggplot(Logf2_db4_eff$treatment, aes(y=fit*100, x=treatment, fill=treatment)) +

geom_bar(color="black", stat="identity", position=position_dodge()) +

geom_errorbar(aes(ymin=lower*100, ymax=upper*100), width=0.2) +

ylab("Prevalence % (predicted GLM)") +

xlab("Treatment") +

ggtitle("Logistf")+

ylim(0, 120)

logf2_db4_Fig + scale_y_continuous(breaks=c(25,50, 75, 100))

# Analysis of infection Intensity (oocyst number)----------------------------------------------------------

#NB Drug_E has no infection (zero prevalence) so oocyst distribution cannot be modelled.

#Remove from data1 to become data1a for these analyses.

#subset data to remove drug_E

data1a<-subset(data1, subset=!(treatment=="drug_E"))

# GLM for intensity, data1a -------------------------------------------------------

#first check model fit to different distributions

#poisson glm on oocyst counts

db4_modinten_poiss<-glm(no_oocysts ~ treatment + bldml_gc + feedno, family=poisson, data=data1a)

summary(db4_modinten_poiss)

#check fit of poisson model

plot(db4_modinten_poiss)

#(overdispersed)

#run glm with quasi-poisson distribution

db4_modinten_qpois<-glm(no_oocysts ~ treatment + bldml_gc + feedno, family=quasipoisson, data=data1a)

summary(db4_modinten_qpois)

#(overdispersed)

#run GLM with negative binomial distribution using R package MASS

db4_modinten_nb1<-glm.nb(no_oocysts ~ treatment + bldml_gc + feedno, data=data1a)

summary(db4_modinten_nb1)

#run GLM with hurdle-negative binomial model using R package pscl

db4_modinten_hurdle0<-hurdle(no_oocysts ~ treatment + bldml_gc + feedno, data=data1a, dist="negbin")

summary(db4_modinten_hurdle0)

#backward elimination of full model : remove non-sig terms (bldml_gc)

db4_modinten_hurdle2<-hurdle(no_oocysts ~ treatment + feedno, data=data1a, dist="negbin")

summary(db4_modinten_hurdle2)

#run GLM with zinb distibution using package pscl

db4_modinten_zinb<-zeroinfl(no_oocysts ~ treatment + bldml_gc + feedno, data=data1a, dist="negbin")

summary(db4_modinten_zinb)

#backward elimination of full model : remove non-significant terms - feedno

db4_modinten_zinb2<-zeroinfl(no_oocysts ~ treatment + bldml_gc, data=data1a, dist="negbin")

summary(db4_modinten_zinb2)

# backward elimination of full model : remove non-significant terms - bldml_gc

db4_modinten_zinb3<-zeroinfl(no_oocysts ~ treatment + feedno, data=data1a, dist="negbin")

summary(db4_modinten_zinb3)

# backward elimination of full model : remove non-significant terms - bldml_gc + feedno

db4_modinten_zinb4<-zeroinfl(no_oocysts ~ treatment, data=data1a, dist="negbin")

summary(db4_modinten_zinb4)

#compare fits of all models: look at regression cofficients

fm2<-list("ML-Pois"=db4_modinten_poiss, "Quasi-Pois"=db4_modinten_qpois, "NB"=db4_modinten_nb1,

"Hurdle-NB"= db4_modinten_hurdle0, "Hurdle-NB-noGC"= db4_modinten_hurdle2,

"ZINB"= db4_modinten_zinb,"ZINB-no_feedno"= db4_modinten_zinb2, "ZINB-noGC"= db4_modinten_zinb3,

"ZINB-Txonly"= db4_modinten_zinb4)

sapply(fm2, function(x) coef(x) [1:8])

rbind(logLik = sapply(fm2, function(x) round(logLik(x), digits=0)),

Df = sapply(fm2, function(x) attr(logLik(x), "df")))

#check how well zero counts are captured

round(c("Obs" = sum(data1a$no_oocysts < 1),

"ML-Pois" = sum(dpois(0, fitted(db4_modinten_poiss))),

"NB" = sum(dnbinom(0, mu=fitted(db4_modinten_nb1), size=db4_modinten_nb1$theta)),

"NB-Hurdle" = sum(predict(db4_modinten_hurdle0, type="prob")[,1]),

"NB-Hurdle-noGC"= sum(predict(db4_modinten_hurdle2, type="prob")[,1]),

"ZINB" = sum(predict(db4_modinten_zinb, type="prob")[,1]),

"ZINB-no_feedno" = sum(predict(db4_modinten_zinb2, type="prob")[,1]),

"ZINB-noGC" = sum(predict(db4_modinten_zinb3, type="prob")[,1]),

"ZINB-Txonly" = sum(predict(db4_modinten_zinb4, type="prob")[,1])))

#check fitted zero components

sapply(fm2[1:8], function(x) round(x$coefficients$zero, digits=3)))

# Conclusion on best fit model based on log likelihood and prediction of zeroes: here ZINB model with no GC

summary(db4_modinten_zinb3)

# predictions of oocyst number in different treatment groups, GLM of best fit model ----------

#for zinb3 (model includes treatment and feedno) using R package ggeffects

summary(db4_modinten_zinb3)

db4_modinten.plot <- predict_response(db4_modinten_zinb3, terms = "treatment", margin = "empirical")

db4_modinten.plot

db4_modinten.plot<-as.data.frame(db4_modinten.plot)

db4_modinten.plot

#draw better graph of predicted results using R package ggplot

db4_intensitymod_Fig<-ggplot(db4_modinten.plot, aes(y=predicted, x=x, fill=x)) +

geom_bar(color="black", stat="identity", position=position_dodge()) +

geom_errorbar(aes(ymin=conf.low, ymax=conf.high), width=0.2) +

ylab("Oocyst number (predicted GLM)") +

xlab("Treatment") +

ylim(0, 30)

db4_intensitymod_Fig + scale_y_continuous(breaks=c(0,5,10,15, 20, 25))

**Supplementary Table 1:** GLM output from best fit model for infection prevalence. Final model included significant effects of Drug and Replicate, but not gametocyte density (not significant, P=0.87).

|  | **Response variable: Infection prevalence** | | | |
| --- | --- | --- | --- | --- |
|  | **GLM model** | | **Logistf model** | |
| **Predictor**  **variable** | **Estimate (standard error)** | **P value** | **Estimate**  **(standard error)** | **P value** |
| Drug A | 0.54 (0.56) | 0.34 | 0.51 (0.54) | 0.35 |
| Drug B | -0.83 (0.46) | 0.07 | -0.79 (0.45) | 0.07 |
| Drug C | -1.60 (0.44) | **0.0003** | -1.54 (0.43) | **0.0001** |
| Drug D | -5.47 (0.81) | **1.6x10^-11^** | -5.16 (0.74) | **0.000** |
| Drug E | -20.54 (816.35) | 0.04 | -6.74 (1.45) | **0.000** |
| Feed 2 | 1.09 (0.38) | **0.004** | 1.06 (0.37) | **0.004** |
| Feed 3 | 0.76 (0.36) | **0.04** | 0.74 (0.35) | 0.04 |
| Constant | 1.25 (0.41) | **0.002** | 1.21 (0.4) | **0.001** |
| Gametocyte density* | -0.013 (0.07) | 0.85 | -0.01 (0.07) | 0.87 |
| N | 401 | | 401 | |
| Log Likelihood | -136.76 | | 270.2 | |
| Akaike Inf. Crit. | 289.53 | | 284.2 | |

*Non-significant, not included in final model

**Supplementary Table 2:** GLM output from best fit model (zero-inflated negative binomial) for oocyst numbers. Final model included significant effects of Drug and Replicate, but not gametocyte density (not significant, P=0.064)

|  | **Response variable: Oocyst number** | | | | |  |
| --- | --- | --- | --- | --- | --- | --- |
| **ZINB GLM model** | **Count model estimate (negbin with log link)** | | **Zero-inflation model estimate (binomial with logit link)** | | |  |
| **Predictor**  **variable** | **Estimate (standard error)** | **P value** | | **Estimate**  **(standard error)** | **P value** | |
| Drug A | -0.45 (0.21) | **0.03** | | -10.41 (529.6) | 0.98 | |
| Drug B | -1.34 (0.22) | **5.1 x 10^-10^** | | -9.70 (398.7) | 0.98 | |
| Drug C | -1.45 (0.26) | **2.3 x 10^-8^** | | 4.81 (2.5) | 0.057 | |
| Drug D | -6.12 (0.79) | **1.1 x 10^-14^** | | 5.54 (4.1) | 0.18 | |
| Feed 2 | 0.18 (0.21) | 0.40 | | -13.23 (106.1) | 0.91 | |
| Feed 3 | -0.33 (0.21) | 0.11 | | -3.97 (2.0) | **0.047** | |
| Constant | 3.01 (0,21) | <2 X 10^-16^ | | -3.25 (2.45) | 0.19 | |
| Log (theta) | -0.30 (0.10) | 0.004 | | n/a | n/a | |
| Gametocyte density* | -0.06 (0.03) | 0.064 | | -0.21(0.26) | 0.41 | |
| Log Likelihood | -839.4 | | | | |  |
| Theta | 0.74 | | | | |  |

*Non-significant, not included in final model
